# Relation between mortality rate, duration of hospitalization and levels of TNFα, IL-6 and catalase at admission of cases to the emergency department with COPD attack

**DOI:** 10.1186/cc9606

**Published:** 2011-03-11

**Authors:** A Bayır, P Büyükünaldı, A Kıyıcı, A Ak, F Kara

**Affiliations:** 1 Selçuk University, Selçuklu Faculty of Medicine, Konya, Turkey; 2 Selçuk University, Meram Faculty of Medicine, Konya, Turkey

## Introduction

The aim of the study was to investigate the relation between the mortality rate, the hospitalization period in the emergency department or ICU and the obtained levels of TNFα, IL-6 and catalase before they underwent attack treatment at admission of the cases applying to the emergency department with COPD attack.

## Methods

The cases diagnosed with COPD before and who applied to the emergency department with COPD attack were included in the study. Venous blood samples were obtained to evaluate the levels of TNFα, IL-6, catalase, leucocyte, sedimentation and CRP when the cases applied to the emergency department. Their hospitalization in the service or ICU, the follow-up period in mechanical ventilation and leaving hospital (dead or discharged) were followed. The mean levels of TNFα, IL-6, catalase, leucocyte, sedimentation and CRP values were compared with the average period of hospitalization in the service or ICU and with each other. The Mann-Whitney U test and chi-square test were used as nonparametric tests. *P *≤ 0.05 values were regarded as significant.

## Results

All of the cases that died (*n *= 7) were followed in intensive care, they underwent invasive mechanical ventilation treatment and their mean hospitalization period was 25 days. The cases discharged (*n *= 80) were all followed in the service and their average hospitalization duration was 6.2 days. Non-invasive mechanical ventilation was applied to 12 of these cases. Of the dead cases, the mean leukocyte value was 12.665, sedimentation 29.68, CRP 49.7, TNFα 27.3, IL-6 32 and catalase was 81. Of the cases discharged, the mean leukocyte value was 8.200, sedimentation 19.0, CRP 49.7, TNFα 29.3, IL 13.6 and catalase was 85.9. The mean value of leukocyte, sedimentation, CRP and IL-6 of the dead cases were significantly higher than those of the cases in the discharged group (*P *= 0.040, 0.038, 0.02, 0.017, respectively).

## Conclusions

A high level of leukocyte, sedimentation, CRP values and low IL-6 values at the admission of cases with COPD attack to the emergency department may indicate the requirement to follow in the ICU and treatment with mechanical ventilation, and a high mortality rate.

